# uPARAP Function in Cutaneous Wound Repair

**DOI:** 10.1371/journal.pone.0092660

**Published:** 2014-03-24

**Authors:** Maryam G. Rohani, Yu-Hua Chow, Maria V. Razumova, Samuel Ash, Chi F. Hung, Lynn M. Schnapp

**Affiliations:** 1 Center for Lung Biology, Division of Pulmonary and Critical Care Medicine, Department of Medicine, University of Washington, Seattle, Washington, United States of America; 2 Department of Bioengineering, University of Washington, Seattle, Washington, United States of America; Boston University Goldman School of Dental Medicine, United States of America

## Abstract

Optimal skin wound healing relies on tight balance between collagen synthesis and degradation in new tissue formation and remodeling phases. The endocytic receptor uPARAP regulates collagen uptake and intracellular degradation. In this study we examined cutaneous wound repair response of uPARAP null (uPARAP^-/-^) mice. Full thickness wounds were created on dorsal surface of uPARAP^-/-^ or their wildtype littermates. Wound healing evaluation was done by macroscopic observation, histology, gene transcription and biochemical analysis at specific intervals. We found that absence of uPARAP delayed re-epithelialization during wound closure, and altered stiffness of the scar tissue. Despite the absence of the uPARAP-mediated intracellular pathway for collagen degradation, there was no difference in total collagen content of the wounds in uPARAP^-/-^ compared to wildtype mice. This suggests in the absence of uPARAP, a compensatory feedback mechanism functions to keep net collagen in balance.

## Introduction

The three classic stages of wound repair include inflammation, new tissue formation and remodeling [1]. During cutaneous wound repair, tissue formation stage occurs about 2–10 days post injury. At this stage migration of epithelial cells contributes to wound closure, and fibroblasts deposit collagen to form granulation tissue beneath the wound [2]. The balance between collagen synthesis and degradation determines net collagen accumulation and thereby scar formation [3]. In addition to the extracellular proteolysis of collagen mediated by matrix metalloproteinases and cysteine cathepsins [4], intracellular proteolysis of collagen occurs through internalization by cell receptors including the macrophage mannose receptor (MRC1) and uPARAP (urokinase plasminogen activator receptor–associated protein) [5], [6], [7].

The receptor uPARAP/Endo180 is a member of macrophage mannose receptor family that is expressed on fibroblasts, macrophages and a subset of endothelial cells [8]. uPARAP is essential for intracellular collagen degradation pathway [9], [10]. uPARAP binds to collagen I, IV and V, which leads to internalization and lysosomal degradation of collagens [11]. Furthermore, uPARAP facilitates migration of fibroblasts on collagen fibrils [9]. Absence of uPARAP leads to excess collagen deposition in matrix in mouse models of lung, kidney and liver fibrosis [12], [13], [14]. Increased expression of uPARAP is associated with tumor progression in several forms of cancer [15], [16] and in a mouse model of malignancy [17]. Although uPARAP is highly expressed in skin [13], its role during wound repair is unknown.

The present study was undertaken to determine the role of uPARAP in cutaneous wound repair. Because of the role of uPARAP in fibroblast migration and collagen degradation, we hypothesized that uPARAP would facilitate wound closure and regulate accumulation of granulation tissue. Our findings demonstrate that absence of uPARAP impairs re-epithelialization process, but its function in collagen turn-over is compensated by other mechanisms during skin wound repair, thus has no major effect on collagen accumulation.

## Methods

### Ethics statement

Experiments were performed under a protocol approved by University of Washington's Institutional Animal Care and Use Committee (permit number 4065-01). All surgical procedures were performed under tribromethanol (avertin 2%) anesthesia, and all efforts were made to minimize suffering.

### Mouse model of excisional wound preparation and analysis

uPARAP^-/-^ mice [9] (FVB) were backcrossed onto C57BL/6 mice for at least eight generations. Age-matched wildtype littermate mice (hereafter referred to as “wildtype”) were used as control. We used a standard method for cutaneous wound model in mice [18]. We used 5-mm biopsy punch (Militex, York, PA) to create four full thickness wounds on the dorsal surface of mice. Subsequently each wound sample was used for histology, analysis of collagen content, biomechanical test or transcription regulation in wound area. Digital photographs of wounds were taken immediately following the excisional biopsy (day 0), and at indicated time points thereafter. The wound area was measured using ImageJ software [19] and percent of wound closure (compared to day 0) was calculated. On indicated days post injury, wounds and their surrounding area were excised with an 8-mm biopsy punch for further analysis.

### Gene expression analysis by quantitative real time-PCR

Skin samples were homogenized in RLT buffer with Omni bead ruptor homogenizer. Total RNA was isolated with RNeasy plus kit (Qiagen, Valencia, CA), according to manufacturer's instruction, and reverse transcribed using High Capacity cDNA Reverse Transcription Kit (Applied Biosystem, Grand Island, NY). PCR was performed using cDNA containing 31 ng RNA. In uPARAP^-/-^ mice, exons 2-6 of uPARAP gene are replaced by an HPRT expression cassette [9], thus we used HPRT as our endogenous control for uPARAP gene expression. For all other genes (MMPs, collagens), we used β2M as our endogenous control. Quantitative real-time PCR was done using ABI7900HT and pre-designed primer and probes sets (ABI TaqMan Gene Expression Assays) for HPRT or β2M (as endogenous controls), and uPARAP, MMP 2, 9, 10, 14, collagen I-α1, III-α1 (target probes). Analysis was done using MS Excel calculating RQ by 2^−ΔΔCT^.

### Histology analysis of wound sites

Wound samples were fixed in 4% formaldehyde buffered in PBS. Paraffin-embedded sections were stained with hematoxylin and eosin (H&E) or Masson's trichrome and digitally scanned (Hamamatsu NanoZoomer) for histology evaluation. For quantification of collagen content in trichrome stained slides we used Visiopharm software (Hørsholm, Denmark), and measured ratio of collagen stained area versus total tissue area. For analysis of re-epithelialization we measured the distance between the edge of the original wound and the leading edge of the re-epithelized area, and presented data as percentage of re-epithelialization [20]. Granulation tissue area was quantitatively evaluated in Masson's trichrome stained samples and defined as the new connective tissue with high cellularity, flanked by dense collagen bundles.

### Gel Contraction Assay

Mouse dermal fibroblasts were isolated as previously described [21], [22]. Cells were maintained in DMEM with 10% FBS, 100 U/ml penicillin, 100 U/ml streptomycin and 5 mM glutamate at 37°C in 5% CO_2_. Studies were performed on cells within 3–6 passages. Wildtype and uPARAP null derived dermal fibroblasts (1.5×10^5^ cells/ml) were incubated in 1 mg/ml type I collagen gels with serum-free media (SFM) alone, PDGF-BB 1.7 nM (R&D Systems), or 10% FBS. To determine gel contraction we followed the standard protocol [23], [24], [25]. Briefly, after one week of incubation at 37°C, collagen gels were fixed by addition of 200 μl formalin solution (37%) to the wells and subsequently incubated for several hours at room temperature. After washing, gels were weighed. Lower gel weights indicate greater contraction, due to less water in the gel.

### Hydroxyproline analysis

The excised wounds were weighed and water was added proportionally for homogenization. Equal volume of homogenized tissue was used for acid hydrolization. Hydroxyproline measurement was done following the manufacturer's instruction (BD Bioscience, San Jose, CA). Hydroxyproline content was extrapolated based on the standard curve generated using known concentration of reagent hydroxyproline. Data are presented as amount (microgram) per mg tissue weight.

### α-SMA protein expression

To assess α-SMA protein expression, wound samples were homogenized in lysis buffer (Sigma, St. Louis, MO) and centrifuged for 10 min at 14000 rpm (at 4°C). Protein concentrations were determined by the BCA assay (Pierce, Rockford, IL). Western blot analysis was done as previously described [26]. Briefly equal amounts of protein were separated by sodium dodecyl sulfate-polyacrylamide gel electrophoresis (SDS-PAGE), and electrophoretically transferred to PVDF membrane. After blocking with 5% nonfat dry milk/0.01% Tween-20/PBS the membrane was incubated with mouse anti-αSMA IgG (1∶10,000) (Sigma), overnight at 4°C. Horseradish peroxidase-conjugated goat anti-mouse IgG (1∶4,000) was used as a secondary antibody. The membrane was then developed with enhanced chemiluminescence (ECL) technique (Pierce, Rockford, IL). We used NIH ImageJ for densitometric analysis of relative band intensities. Values were normalized to GAPDH control and presented as relative intensities compared to samples from wildtype mice.

Immunohistochemical detection of α-SMA was performed on formalin fixed, paraffin embedded tissues with mouse anti-αSMA IgG (1∶200) (Sigma) and the Mouse on Mouse Elite peroxidase kit (Vector Laboratories, Burlingame, CA) following manufacturer's instruction. We included a negative control (no primary antibody) for each sample. Visiopharm software was used for quantification of area positive for α-SMA in the extent of granulation tissue area.

### Mechanical measurements

Skin patches from uninjured areas or healed wounds of the same area, on day 12, were dissected along the direction of hair growth into strips and suspended on stainless steel hooks attached to a force transducer (Aurora Scientific, model 400A) and a length controller (Aurora Scientific, model 308B). We determined the stiffness (dF/dL) by imposing change in length of the skin strip and measured the resultant change in force. The dimensions of strips tested were 2.2±0.5 mm by 730±140 μm (L x W). On each strip of tissue, from the initial non-stretched length, 9 steps of 5% length stretches were taken at intervals of 30 seconds. The test was done on at least three strips from each mice and the average was calculated. Force and length signals were digitally recorded and analyzed using custom LabView software. To normalize between preparations, force was divided by the width of the strip. All measurements were acquired in PBS solutions at room temperature. Data are presented as the amount of force change per millimeter of length change.

### Statistical Analysis

Statistical analysis was performed using the Graphpad Prism (version 5). We used t-test to compare two groups, or two-way ANOVA followed by bonferroni post-test to test the effect of two factors. A p-value less than 0.05 was considered statistically significant.

## Results

### uPARAP expression increases during cutaneous wound healing

**Figure 1:**
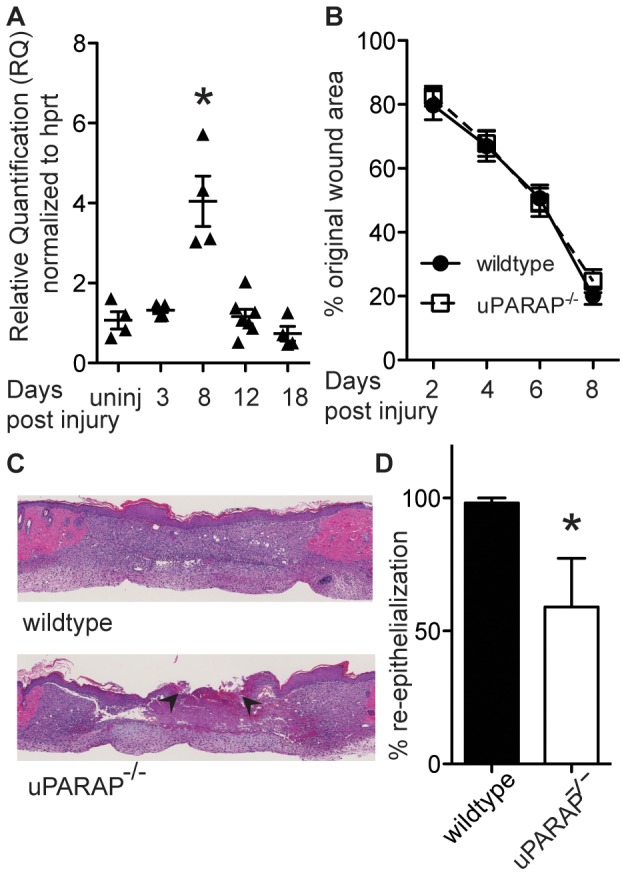
Expression of uPARAP during skin wound repair. (A) Wound samples were collected on indicated days post- injury. mRNA transcription was measured using quantitative real time-PCR. Data were normalized to HPRT expression. *Y axis* represents fold change relative to uninjured skin. Each point represents an individual mouse. * *P* <0.05, compared to uninjured skin. **Effect of uPARAP on wound closure.** (B) On indicated days post-injury, photographs of wounds were taken and percent of wound closure was measured compared to the original wound area using ImageJ analysis (n = 14 wildtype, 13 uPARAP^-/-^). (C) H&E staining of wound sections on day 8 post-injury shows incomplete re-epithelialization in uPARAP^-/-^ mice. Arrowheads mark the edges of the wound. Section through the midpoint of the wound in wildtype animal shows complete re-epithelialization. (D) Histology assessments of re-epithelialization were quantified (n = 5 wildtype, 4 uPARAP^-/-^ mice), * *P*<0.05.

uPARAP is highly expressed in the normal mouse skin [13], [27]. We examined uPARAP mRNA expression following full thickness excisional biopsy and subsequent wound healing in wildtype mice. We found that in skin from uninjured wildtype mice, the Ct range was between 26–28, indicating relevantly high level of uPARAP expression at baseline. After the initial wound, uPARAP expression increased and reached its maximum level on day 8 post-injury, and then returned to baseline by day 18 post-injury (Fig. 1A).

### Absence of uPARAP delays re-epithelialization

Given the upregulation of uPARAP during wound healing, and its role in matrix remodeling, we asked whether absence of uPARAP would impair wound healing. We measured the kinetics of wound closure in wildtype and uPARAP^-/-^ mice. Macroscopic measurements showed no significant difference between the two genotypes at any of the time post-wounding (Fig. 1B). However, while all wildtype mice had complete re-epithelialization of wounds by day 8 by histological evaluation, uPARAP^-/-^ mice had significantly incomplete re-epithelialization of wounds (Fig. 1C, D). By day 12 post-injury, both wildtype and uPARAP^-/-^ mice showed complete re-epithelialization (data not shown). Thus absence of uPARAP delays the normal re-epithelialization.

**Figure 2:**
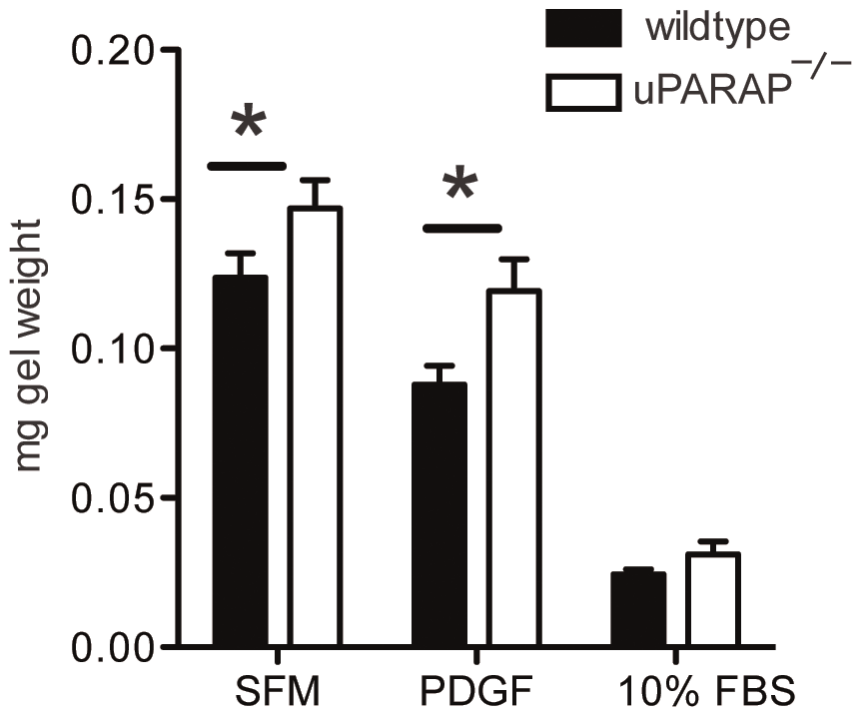
Collagen contraction of uPARAP^-/-^ dermal fibroblasts. Dermal fibroblasts from wildtype or uPARAP^-/-^ mice were grown in collagen gels with serum-free media, PDGF-BB, or 10% FBS for one week, then collagen gels were weighed. Lower weight indicates greater gel contraction. * *P*<0.05 compared to wildtype. (n = 3/group)

In mice, contraction accounts for a large part of wound closure [28]. Therefore we tested ability of dermal fibroblasts from wildtype and uPARAP^-/-^ mice to contract collagen matrix. We found that dermal fibroblasts from uPARAP^-/-^ showed decreased contractility compared to wildtype mice at baseline, and after stimulation with PDGF (p<0.05) (Fig. 2).

### Collagen turnover is modulated in the absence of uPARAP

**Figure 3:**
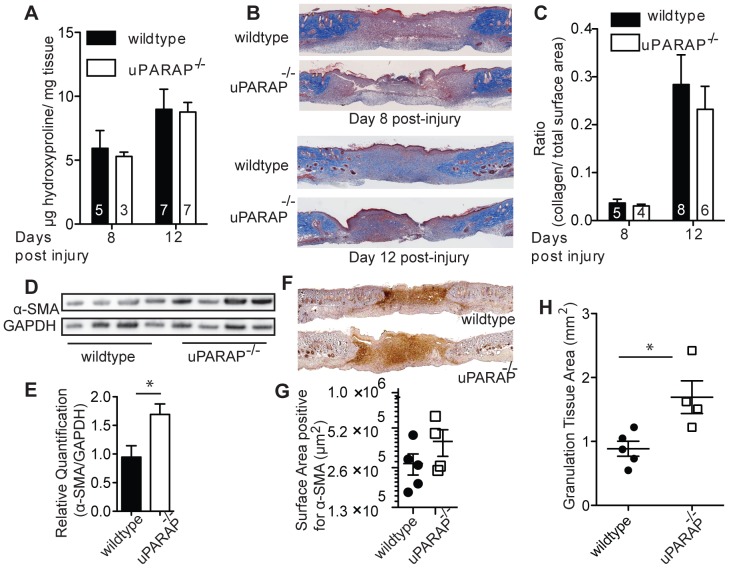
Collagen content in wildtype and uPARAP^-/-^ after wound injury. (A) Hydroxyproline content was measured in wounds on day 8 and 12 post-injury. (B) Trichrome staining was done on fixed wound samples on day 8 and 12 after wounding. Representative images are shown from at least 4 mice/genotype (C) Ratio of collagen staining area versus total tissue in wound area was quantified in slides stained with trichrome. Data are presented as mean± SEM; n of mice in each group is indicated inside the bars. (D) α-SMA expression in wound tissue homogenates from day 8 post-injury was evaluated by Western blot analysis (top). GAPDH was used as a loading control (bottom), n = 4/genotype. (E) Intensity of bands on immunoblots were quantified and normalized to GAPDH. (F) Representative images of α-SMA staining on fixed wound samples on day 8 post-injury. (G) The area positive for α-SMA was quantified. Each point represents an individual mouse. (H) Area of granulation tissue was traced and quantified in trichrome stained wound samples from day 8. Data are presented as mean± SEM, * *P*<0.05 compared to wildtype.

Since uPARAP mediates intracellular collagen degradation [9], [29], we hypothesized that uPARAP^-/-^ mice would have increased collagen accumulation during the wound repair process. However, hydroxyproline analysis showed no difference between wildtype and uPARAP^-/-^ mice at day 8 or 12 post-injury (Fig. 3A). Similarly no significant difference was observed in trichrome staining of skin sections on day 8 or 12 after wounding (Fig. 3B, C). This suggested the presence of compensatory mechanism(s) such as decreased collagen synthesis or increased collagen degradation by other pathways (ie MMPs) in uPARAP^-/-^ mice in order to keep total collagen content unchanged.

**Figure 4:**
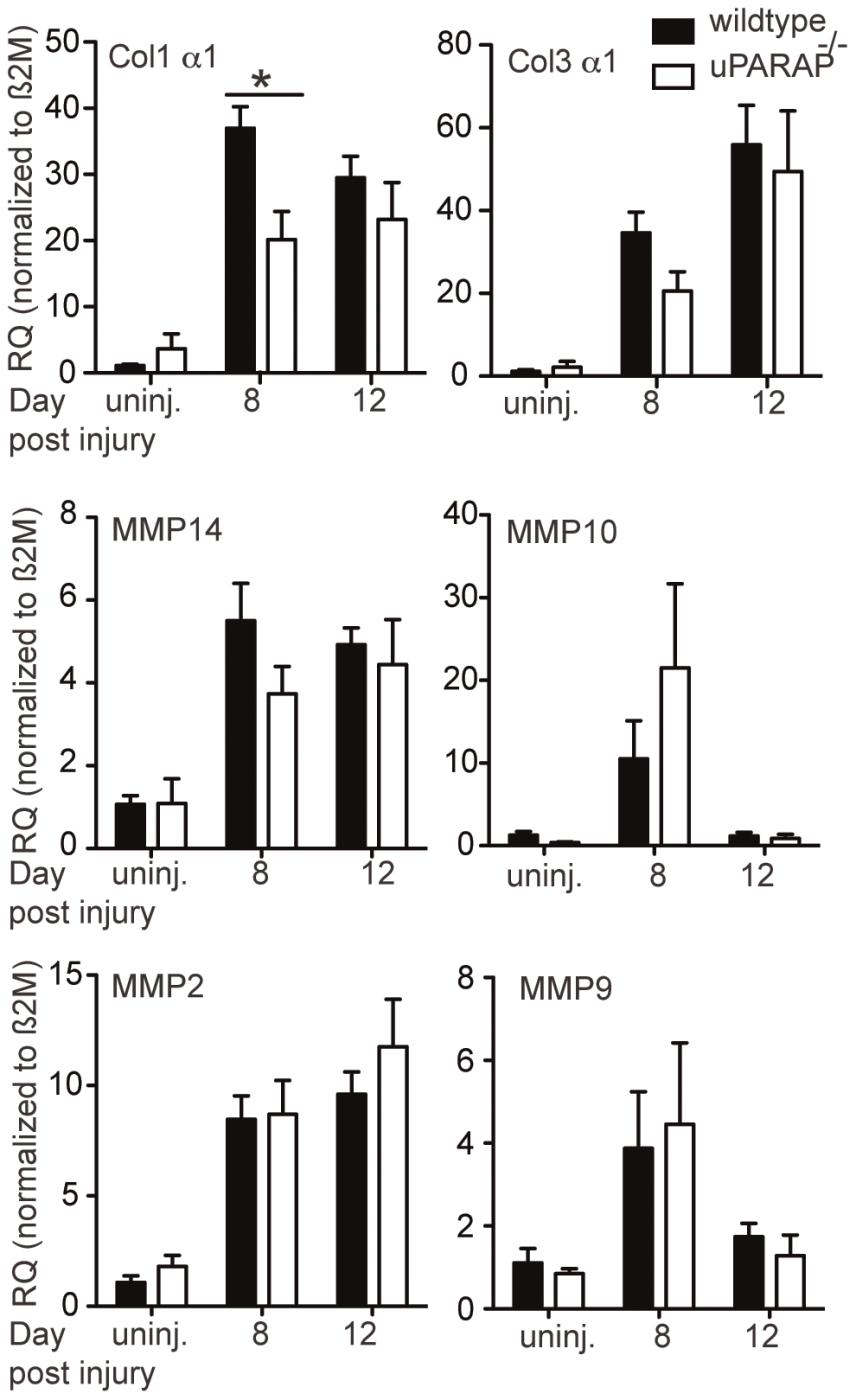
Gene expression analysis. Total RNA was extracted from intact skin or wound samples on day 8 or 12 post-injury. cDNA was made and transcription of target genes was quantified using quantitative real time-PCR method. Data are normalized to β2M housekeeping gene. Data are presented relative to expression in intact skin from wildtype group. * *P*<0.05, n≥3.

We tested the mRNA expression of collagen I and III, the two most ubiquitous collagens in the skin. We found significant decrease of collagen I mRNA in uPARAP^-/-^ compared to their wildtype littermate on day 8 post-injury. By day 12 there was no longer a difference between wildtype and uPARAP^-/-^ mice. Likewise, we found a decrease in collagen III mRNA on day 8, although this did not reach statistical significance (Fig. 4). Since myofibroblasts are the main collagen-producing cells, we examined differences in expression of α-SMA, a marker of myofibroblasts [30] in wound samples on day 8 post injury. We found increased α-SMA expression in uPARAP^-/-^ compared to wildtype mice by western blot analysis (Fig. 3D,E), and by immunohistochemistry (Fig. 3F, G). In addition, we also found larger granulation area larger granulation area in uPARAP^-/-^ mice (Fig. 3H). These data suggest that the decreased collagen mRNA synthesis is not due to a decrease number of myofibroblasts.

We also examined the mRNA expression of MMPs, the main mediators of extracellular collagen proteolysis. MMP14 is a critical collagenolysin in pericellular collagen degradation pathway [31], and its function is important in connective tissue degradation [32]. However, we found no significant difference in expression of MMP14 between wildtype and uPARAP^-/-^ mice. Similarly we found no significant differences in MMP2, MMP9 and MMP10 expression between the two genotypes (Fig. 4).

### Wildtype and uPARAP^-/-^ show different mechanical stability of healing wounds

**Figure 5:**
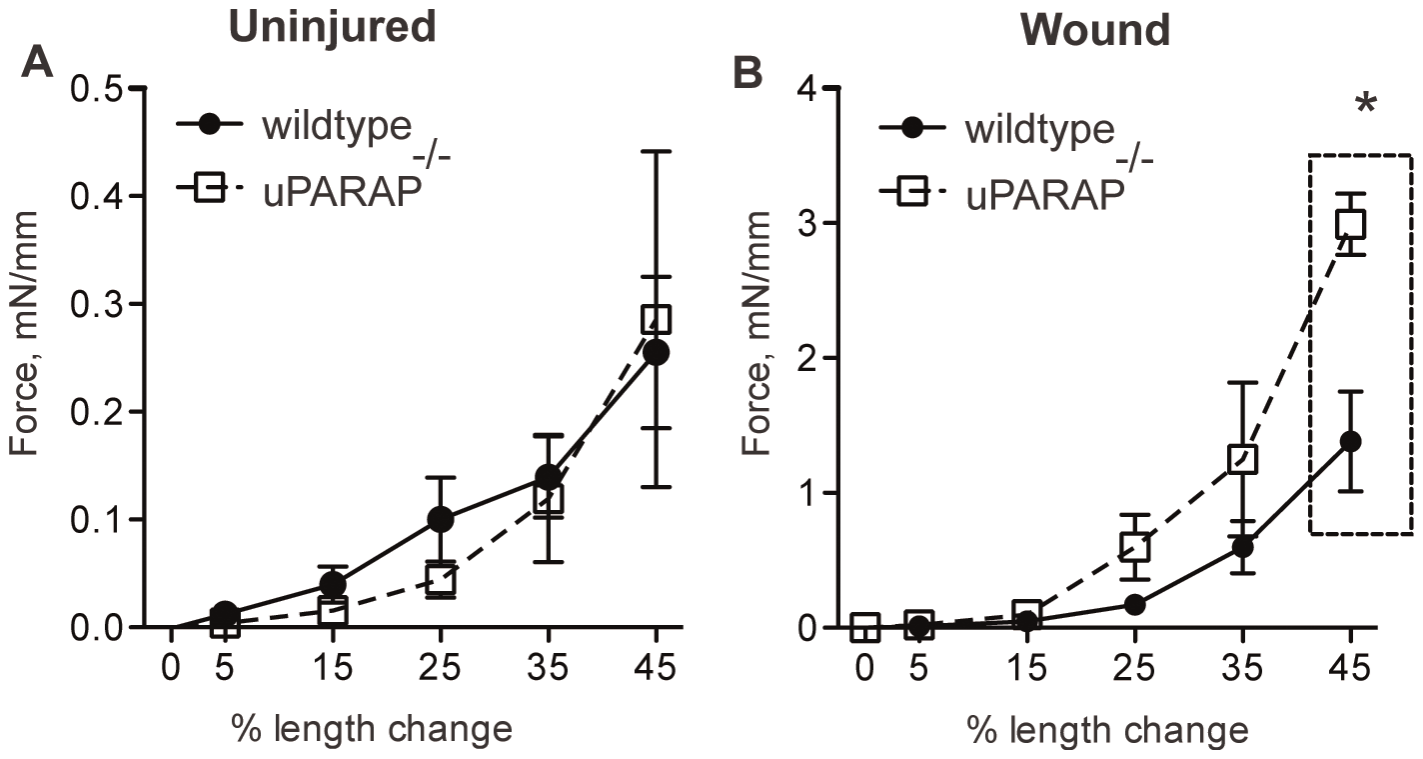
Comparison of stiffness of normal and healed skin in wildtype and uPARAP^-/-^ mice. (A) Full thickness biopsies collected from intact dorsal surface of wildtype and uPARAP^-/-^ mice were analyzed for mechanical characteristics. n = 6/genotype. (B) Skin wounds on day 12 post-wounding (from the same area as intact skin) were tested for tissue stiffness. * *P*<0.05, n = 3/genotype.

To determine whether healed wounds from wildtype and uPARAP^-/-^ have differential tissue mechanical characteristics, we compared stiffness between the two genotypes in intact skin and on day 12 post-injury. At baseline, we did not see any significant difference in intact skin between the two groups (Fig. 5A), but when we compared stiffness of healed wounds between the two genotypes we found that in the absence of uPARAP, tissue stiffness increased significantly (Fig. 5B).

## Discussion

We found time-dependent increased expression of uPARAP during cutaneous wound repair. Its absence delayed re-epithelialization process, and altered physical strength of repaired wound, possibly via changes in composition or structure of extracellular matrix. However, we did not observe any difference in collagen content between wildtype and uPARAP^-/-^ mice.

During wound healing, fibroblasts migrate into the wound area, where they deposit matrix proteins such as collagen and fibronectin to form granulation tissue. Once activated into α-SMA-expressing myofibroblasts, they generate the adhesive and tensile forces required for wound closure [33], [34]. Results from our study showed in the absence of uPARAP, there is a delay in reconstruction of epidermis. Epithelial cells do not express uPARAP [8], [17]. Thus the observed phenotype of delayed re-epithelialization could be the result of decreased contraction in wound bed of uPARAP^-/-^ mice. This hypothesis is supported by our in vitro study on dermal fibroblasts, which showed that cells from uPARAP^-/-^ mice have less contractile ability compared to wildtype mice, as measured in collagen gel contraction assay. Lack of differences in MMP expression suggests that the results are due to contraction of gel, rather than degradation of the gel. Alternative explanations for delay in re-epithelialization include changes in microenvironment such as modifications in extracellular matrix composition and arrangement, which consequently cause impairment in epithelial cell migration.

Based on the known function of uPARAP in collagen internalization and prior studies demonstrating increased collagen in several model of fibrosis, we expected to see increased collagen accumulation in uPARAP^-/-^ during wound healing. Surprisingly, we did not find any difference in collagen between wildtype and uPARAP^-/-^ mice, although our study was underpowered to detect small differences in collagen content. Unappreciated baseline differences in collagen content between the genotypes could mask subsequent changes in collagen remodeling after injury. Additional explanations include upregulation of other pathways for collagen degradation or decreased collagen synthesis. Our data demonstrate decreased collagen expression in the absence of uPARAP, but no significant difference in expression of MMPs in uPARAP^-/-^ mice. The lack of difference in MMP expression is consistent with our prior reports examining MMP expression during lung development and during lung injury in wildtype and uPARAP^-/-^ mice [13], [35]. The decrease in collagen mRNA expression was measured from total wound tissue, which includes a variety of cell types. The major collagen-synthesizing cell type during wound repair is the α-SMA-expressing myofibroblast. Interestingly, α-SMA expression was increased in uPARAP^-/-^ wounds (Fig. 3D–F), suggesting that the decreased collagen expression was less likely caused by decreased number of fibroblasts. We speculate that decreased endocytosis of proteolysed collagen in uPARAP^-/-^ mice, leads to increased accumulation of fragmented collagen in extracellular environment, as seen in other studies [3], [36], [37]. Studies by Verani *et al* showed that accumulation of extracellular type I collagen fragments resulted in decreased type I procollagen synthesis by skin fibroblasts, although the mechanism is unknown. [38], [39]. We propose a similar feedback loop: absence of uPARAP-mediated endocytosis leads to accumulation of collagen fragments, which leads to decreased collagen synthesis during wound healing.

Our findings showed that in uPARAP^-/-^ skin stiffness is increased after wound healing. Composition of ECM, and more specifically collagen organization in skin dermis is a critical determinant of mechanical strength. In arteries, increased ratio of collagen degradation to collagen synthesis causes increased stiffness [40]. In our study, unchanged total collagen content, in combination with decreased expression of collagen I in uPARAP^-/-^ mice suggests differences in maturity of collagen matrix, such as differences in organization and cross-linking which affect mechanical properties [41]. In addition, we also found increased α-SMA expression in wounds, which may also contribute to altered mechanics. In contrast to our findings in the lungs of uPARAP^-/-^ mice, in which we found decreased lung compliance both at baseline and after lung injury [13], we only found differences in skin stiffness after injury. However, it is possible that other techniques to measure mechanical properties of the skin such as bursting strength assay may yield differences at baseline.

Collectively, we found that uPARAP is up-regulated during wound healing, and absence of uPARAP resulted in delayed re-epithelialization, decreased collagen mRNA expression and altered wound mechanics. We conclude that uPARAP is an important modulatory receptor during wound repair that facilitates proper wound closure and provisional matrix remodeling.
